# Investigation into the Strength, Hydration, and Microstructural Characteristics of Clinker-Free Cement Composed of Phosphorus Slag, Fluidized Bed Combustion Bottom Ash, and Lime

**DOI:** 10.3390/ma18143266

**Published:** 2025-07-10

**Authors:** Yanzhou Peng, Haitian Li, Hefei Yin, Ji Xiao, Gang Xu

**Affiliations:** 1 Hubei Key Laboratory of Disaster Prevention and Mitigation, China Three Gorges University, Yichang 443002, China; postxg@163.com; 2 College of Civil Engineering and Architecture, China Three Gorges University, Yichang 443002, China; 13681221527@163.com (H.L.); yinalbert628@gmail.com (H.Y.); qq375607219@126.com (J.X.); 3 Second Harbor Engineering Company Ltd., Wuhan 430040, China

**Keywords:** phosphorus slag, fluidized bed combustion bottom ash, alkali-activated materials, strength, durability, microstructure

## Abstract

This study focuses on developing a novel clinker-free cement, specifically comprising phosphorus slag-based cementitious materials (PSCMs), by utilizing lime and industrial byproducts, including granulated electric furnace phosphorus slag and fluidized bed combustion bottom ash. The optimal composition of PSCM was determined by investigating the effects of different proportions of activators (water glass and sodium sulfate) and retarder (potassium fluoride) on the setting time and the mechanical strength of PSCMs. Performance evaluations demonstrated that the compressive and flexural strengths of the optimal PSCM formulation at 28 days were 64.1 MPa and 7.5 MPa, respectively. Notably, concrete prepared with the optimal PSCM exhibited superior freeze–thaw resistance and sulfate resistance compared to Portland cement concrete of equivalent strength grades. The comprehensive characterization of selected PSCM compositions, conducted using X-ray diffraction (XRD), Fourier transform infrared spectroscopy (FTIR), thermogravimetric analysis (TGA), and scanning electron microscope–energy-dispersive spectrometry (SEM-EDS), provided in-depth insights into the interrelationship among mechanical properties, durability, and microstructural characteristics. SEM-EDS analysis confirmed that calcium aluminosilicate hydrate and sodium aluminosilicate hydrate are the predominant hydration products of PSCMs. FTIR and TG analyses elucidated the continuous hydration behavior of PSCMs during the curing process, while SEM observations revealed a densely compact microstructure in the hardened PSCM paste.

## 1. Introduction

The growing global population and ongoing urbanization are expected to boost the construction industry, implying there will be consistent demand for building materials such as cement in the foreseeable future. Over the past decade, global cement production has consistently exceeded 4 billion tonnes per year, leading to significant environmental challenges such as carbon dioxide emissions and high energy consumption. Portland cement production, which requires the calcining of limestone at high temperatures, accounts for about 7–8% of global CO_2_ emissions [1,2,3]. Approximately 50–60% of these emissions come from the calcination reaction (a key step in the production process), around 35–40% come from fossil fuel combustion, and come 10–15% from activities such as transportation, space heating, and plant utilities [3,4,5].

To mitigate CO_2_ emissions in cement production, a viable approach is to use industrial solid waste as a partial substitute for cement clinker. This method enables the production of low-clinker cement with comparable or enhanced strength and durability [6,7]. Additionally, using industrial solid waste in clinker-free cement significantly reduces CO_2_ emissions and energy consumption. Supplementary cementitious materials (SCMs) such as fly ash (FA), ground granulated blast furnace slag (GGBFS), steel slag, and metakaolin have demonstrated effectiveness in producing clinker-free cement, and are increasingly accepted in the construction market [8,9,10,11,12,13]. However, the availability of these SCMs, particularly FA and GGBFS, depends on their upstream industries, which may decline in quantity and quality as these industries evolve. Therefore, to support the low-carbon and sustainable development of the cement industry, it is imperative to explore new sources of SCMs for low-clinker or clinker-free cement production.

Granulated electric furnace phosphorus slag (PS), a byproduct of yellow phosphorus production, is generated at a ratio of 8–10 tonnes of PS per tonne of yellow phosphorus [14,15]. PS primarily consists of SiO_2_ and CaO, with an amorphous glass phase content reaching up to 85% by weight, which confers significant pozzolanic reactivity suitable for cementitious materials [16,17,18,19]. However, the low Al_2_O_3_ content and high SiO_2_ concentration limit its early reactivity [20,21,22,23,24,25,26]. Moreover, the presence of P_2_O_5_ and F in PS retards the hydration of Portland cement, adversely affecting early strength development [27,28]. Consequently, the utilization of PS remains limited, resulting in substantial stockpiles and the environmental contamination of soil and water. Therefore, further research into effective utilization strategies for PS is urgently required.

Significant efforts have been undertaken to enhance the reactivity of phosphorus slag (PS) through increasing its specific surface area, elevating curing temperatures (including steam curing), and incorporating chemical activators [29,30,31,32]. These approaches have not only improved PS utilization, but also promoted resource conservation and environmental protection. However, challenges persist, particularly concerning the high energy consumption during grinding and the elevated costs associated with high-temperature curing.

Research findings [33,34,35,36] indicate that aluminosilicate materials such as fly ash, GGBFS, and metakaolin, when combined with alkaline components, can produce alkali-activated materials (AAMs), characterized by low carbon emissions and reduced energy consumption. These AAMs exhibit superior chemical and freeze–thaw resistance compared to Portland cement [37,38,39,40], positioning them as promising energy-efficient and environmentally friendly building materials [8,9,10,11,12,13,41]. Phosphorus slag can also be utilized to produce AAMs [42,43,44,45,46]. Extensive studies have explored the development and application of these PS-based AAMs, yielding significant results [47,48]. However, research on alkali-activated PS materials highlights several challenges [18,20,22,24,41,47,49]. First, their production often requires blending PS with Portland cement, GGBFS, fly ash, or metakaolin, leading to a relatively low proportion of PS. Second, concerning fluidized bed combustion bottom ash (BA), the presence of which in emissions has exhibited a steady increase over recent years [50], there are currently no public reports regarding its application in the preparation of alkali-activated materials (AAMs).

This study proposes a novel clinker-free cement composed of phosphorus slag-based cementitious material (PSCM), developed by utilizing lime and industrial solid wastes, including granulated electric furnace phosphorus slag (PS) and fluidized bed combustion bottom ash (BA). This approach promotes the recycling and reuse of these materials. The effects of varying dosages of activators (water glass and sodium sulfate) and retarder (potassium fluoride) on the PSCM’s properties (setting time and strength) were first investigated to determine the optimal composition. Additionally, the sulfate and freeze–thaw resistances of PSCM concrete were examined. Finally, the hydration and microstructure of selected PSCM compositions were analyzed using X-ray diffraction (XRD), Fourier transform infrared spectroscopy (FTIR), thermogravimetry (TG), and scanning electron microscopy (SEM). This paper aims to achieve the following objectives:

(1) To develop PSCMs with a strength grade of 42.5 using phosphorus slag and fluidized bed combustion bottom ash;
(2) To evaluate the freeze–thaw resistance and sulfate resistance of concrete prepared with the developed PSCMs;
(3) To investigate the relationship between the performance and microstructure of the hardened paste of the developed PSCMs through microstructural analysis.

## 2. Experimental Programs

### 2.1. Materials

**Figure 1:**
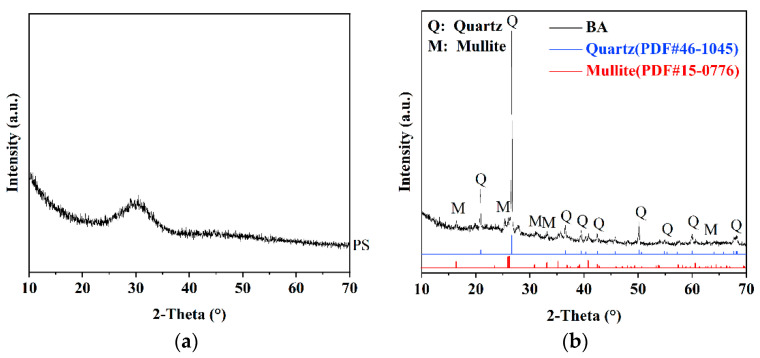
XRD patterns of industrial solid waste used in this study: (**a**) ground granulated electric furnace phosphorus slag (PS), and (**b**) fluidized bed combustion bottom ash (BA).

Ground granulated electric furnace phosphorus slag (PS), fluidized bed combustion bottom ash (BA), and lime (L) were utilized as the primary ingredients of phosphorus slag-based cementitious materials (PSCMs) in this study. The materials used in this study are as follows: Granulated electric furnace phosphorus slag (PS), produced by Hubei Yatai Chemical Co., Ltd. (Yichang, China), has a specific surface area of 397 m^2^/kg. Circulating fluidized bed combustion bottom ash (BA), supplied by Hubei Yihua Group Co., Ltd. (Yichang, China), is of the low-sulfur type and has a specific surface aera of 579 m^2^/kg. Lime (L) was provided by Hubei Yichang Runchu Building Materials Co., Ltd. (Yichang, China). The slaking time and slaking temperature of the lime are 7 min and 60 °C, respectively. The chemical compositions are detailed in Table 1, and the XRD patterns of PS powder and BA are shown in Figure 1. As illustrated in Figure 1, the amorphous glass phase is the primary mineralogical component of PS, indicating its high reactivity [18,27,37]. In contrast, BA is primarily composed of quartz with a minor amount of mullite. ISO standard silica sand, compliant with Chinese Standard GB/T 17671-2021 [51], was used to prepare test specimens for evaluating PSCM performance. River sand with a fineness modulus of 2.5 and crushed limestone (5–20 mm) served as aggregates in the PSCM concrete.

Meanwhile, water glass and sodium sulfate were employed as activators for PSCMs. The water glass used was a commercially sourced aqueous solution with a modulus of 1.2, whereas the sodium sulfate was a commercially available analytical reagent. Furthermore, potassium fluoride (KF) of analytical grade was added as a retarder to modulate the setting time of alkali-activated PSCMs.

### 2.2. Mix Proportions and Samples Preparation

The formulations of the PSCM mixtures considered in this study are tabulated in Table 2.

PSCM paste, mortar, and concrete were prepared according to the formulations presented in Table 2. A 5 L capacity mortar mixer was utilized to mix the PSCM mortar with a water-to-binder ratio (W/B) of 0.5, including water from the activator solution, and a sand-to-binder ratio of 3. Cementitious components (PS, BA, and L) and ISO standard silica sand were initially mixed for 30 s. The alkaline activator solution and mixing water were then gradually added until a uniform mortar was achieved. Fresh mortar was immediately cast into prisms measuring 40 mm × 40 mm × 160 mm and covered with plastic film to prevent moisture evaporation. After 24 h, the specimens were demolded and cured at a temperature of 20 ± 2 °C and a relative humidity of 95% until they reached the designated testing age.

The preparation procedure for the PSCM paste assessed for setting time closely followed that of the PSCM mortar. For the PSCM concrete samples the mix proportions of which are shown in Table 3, all components—including cementitious components (PS, BA, and L), aggregates (river sand and crushed limestone), retarder, alkaline activator solution, and mixing water—were mixed using a 60 L mixer. The thoroughly mixed PSCM was cast and vibrated as per conventional concrete practices. Cubic specimens (150 mm × 150 mm × 150 mm and 100 mm × 100 mm × 100 mm) as well as the prismatic specimen (100 mm × 100 mm × 400 mm) were prepared to evaluate compressive strength, sulfate attack resistance, and freeze–thaw resistance, respectively. After demolding at for 24 h, these specimens were cured at 20 ± 2 °C and 95% until testing.

### 2.3. Methods

**Figure 2:**
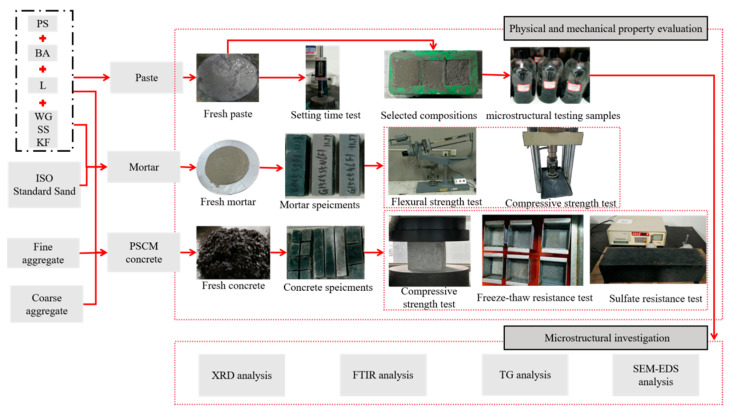
Testing methodology of study.

Figure 2 illustrates the testing methodology employed in this study.

#### 2.3.1. Determination of the Setting Time and Strength of PSCMs

The setting time of PSCMs was determined using a standard consistency paste in accordance with GB/T 1346-2024 [52], utilizing a Vicat apparatus equipped with a needle designed to penetrate the samples with paste at regular intervals.

The strength of PSCMs was evaluated using prismatic mortar specimens measuring 40 mm × 40 mm × 160 mm. These prismatic specimens were prepared as described in Section 2.2. The flexural and compressive strength were measured at 3 and 28 days, following the testing protocol prescribed by GB/T 17671-2021 [51]. Specifically, the flexural strength was calculated as the average of the measurements obtained from three prism specimens (40 mm × 40 mm × 160 mm), while the compressive strength was determined as the arithmetic mean of the results for six specimens.

#### 2.3.2. Determination of PSCM Concrete Performance

The compressive strength of PSCM concrete, with mix proportions as detailed in Table 3, was evaluated at 3 and 28 days according to GB/T 50081-2019 [53]. Additionally, tests for sulfate attack resistance and freeze–thaw resistance were conducted following the methods specified in GB 50082-2024 [54], namely, the Test Method for Rapid Freezing and Thawing and the Test Method for Resistance of Concrete to Sulfate Attack.

#### 2.3.3. Microstructure Examination

The mixtures BA0-WG7-KF1, BA0-WG7-SS3-KF1, BA15-WG7-KF1, and BA15-WG7-SS3-KF1 of the PSCM paste, as presented in Table 2, were selected for microstructural examination. Cubic specimens with dimensions of 40 mm × 40 mm × 40 mm were prepared according to the formulations shown in the table, using a water-to-binder ratio (W/B) of 0.30.

For each mixture, fresh paste was cast into cubic molds and immediately covered with plastic film to prevent moisture loss. After 24 h, the specimens were demolded and subsequently cured at 20 ± 2 °C and 95% until the ages of 3 days and 28 days. Samples were taken for microstructural investigation from the central sections of the cubic specimens at the designated testing age. These samples were then immersed in ethanol for at least 24 h to facilitate dehydration and subsequently dried in an oven at 40 °C for an additional 24 h. Samples intended for XRD, FTIR, and TG-DTG analysis were ground to fine powders with particle sizes less than 10 μm, while those prepared for SEM observations were trimmed to approximate dimensions of 3 mm × 3 mm × 1 mm.

XRD analysis was conducted using an AXS D8-Advance X-ray diffractometer (Bruker, Karlsruhe, Germany) with a scan range of 5° to 70° (2θ) and a step size of 0.02°. FTIR analysis was performed utilizing a Thermo Nicolet Fourier transform infrared spectroscopy (PerkinElmer, Waltham, MA, USA), covering a wavenumber range from 4000 cm^−1^ to 400 cm^−1^. Thermogravimetric analysis (TGA) was conducted using a Netzsch STA 449F3 thermogravimetric analyzer (Netzsch, Bavaria, Germany) under an air atmosphere with the temperature increasing from 20 °C to 1000 °C at a heating rate of 3 °C/min. SEM-EDS analysis was performed using a JSM-IT800 environmental scanning electron microscope (JEOL, Tokyo, Japan) at an acceleration voltage of 20 kV.

## 3. Experimental Results and Discussion

### 3.1. Effect of Activator on the Strength of PSCM

Based on previous research [28], two formulations of clinker-free phosphorus slag-based cementitious materials (PSCMs) were selected: BA0 (0% BA, 5% lime) and BA15 (15% BA, 5% lime). This section experimentally investigates the effects of water glass (WG) and sodium sulfate (SS) dosages on the compressive and flexural strength of PSCMs. WG dosages, defined as the mass ratio of Na2O from WG to the total cementitious materials (PS + BA + L), were set at 5%, 6%, 7%, and 8%. SS dosages, expressed as the mass ratio of SS to the total cementitious materials, were set at 1%, 3%, and 5%. Detailed formulations are provided in Table 2.

#### 3.1.1. Effect of the Water Glass Dosage

**Figure 3:**
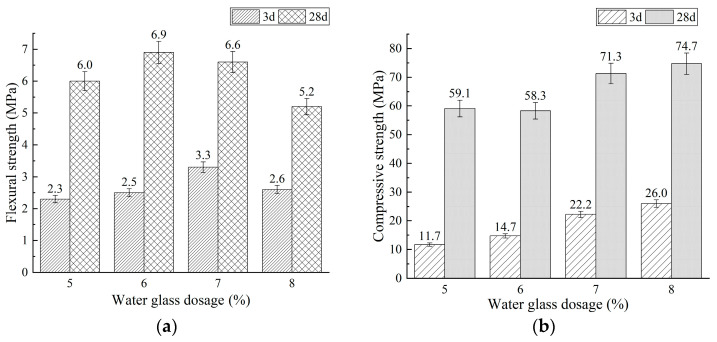
Effect of water glass dosage on strength of mixture BA0: (**a**) flexural strength and (**b**) compressive strength.

**Figure 4:**
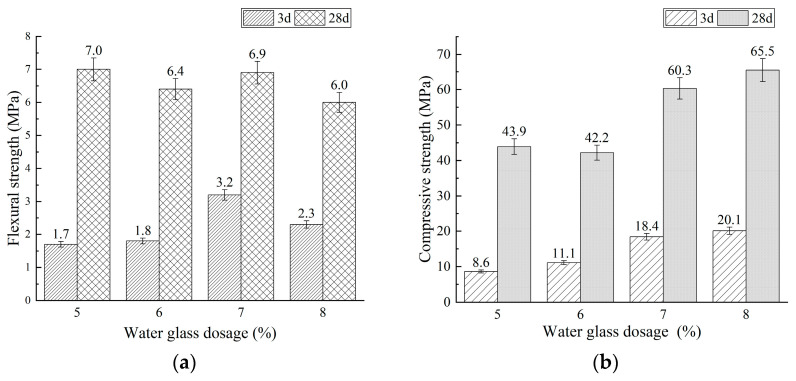
Effect of water glass dosage on strength of mixture BA15: (**a**) flexural strength and (**b**) compressive strength.

The flexural and compressive strengths of mixtures BA0 and BA15, with water glass dosages of 5%, 6%, 7%, and 8%, are diagrammed in Figure 3 and Figure 4, respectively.

As shown in Figure 3 and Figure 4, increasing the water glass dosage from 5% to 8% resulted in a consistent increase in compressive strength for both BA0 and BA15 PSCM mixtures (Figure 3b and Figure 4b). Flexural strength initially increased but then decreased (Figure 3a and Figure 4a). Notably, at a 7% water glass dosage, the 3-day flexural strengths of BA0 and BA15 reached peak values of 3.3 MPa and 3.2 MPa, respectively. In fact, the formation of alkali-activated materials involves the geopolymerization of aluminosilicate constituents in raw materials (precursors) at room temperature or high temperatures [55,56,57,58,59]. This process can be divided into three stages: the depolymerization stage, the repolymerization stage, and the hardening stage [56,57,58,59]. Specifically, the steps are as follows: (1) Aluminosilicate components in precursor are dissolved in the alkaline activator solution, releasing free silicon–oxygen and aluminum–oxygen tetrahedral units. (2) These tetrahedral units repolymerize through hydroxyl condensation reactions, and (3) the silicoaluminate network structure gradually hardens, forming calcium aluminosilicate hydrate (C-A-S-H) and sodium aluminosilicate hydrate (N-A-S-H) gels. Therefore, higher water glass dosages increased the concentrations of OH^−^, silicon–oxygen tetrahedral units, and aluminum–oxygen tetrahedral units, thereby promoting the geopolymerization reactions of phosphorus slag-based materials. This enhancement significantly improved mechanical properties, particularly compressive strength.

Furthermore, Figure 4 shows that under identical water glass dosage conditions, mixture BA15 exhibits lower 3-day flexural and compressive strengths, as well as 28-day compressive strength, compared to mixture BA0. This indicates that incorporating fluidized bed combustion bottom ash (BA) reduces the early-age strength of alkali-activated phosphorus slag-based materials. This phenomenon is closely associated with the chemical compositions of BA and PS: BA is a low-calcium silicon–aluminum material, while PS is high-calcium (Table 1). Generally, alkali-activated high-calcium silicon–aluminum materials, such as ground granulated blast furnace slag (GGBFS), primarily form C-A-S-H hydration products with high calcium contents. In contrast, alkali-activated low-calcium silicon–aluminum materials, including metakaolin and fly ash, predominantly generate N-A-S-H-based products [55,56,60]. At room temperature, the reaction rate of alkali activation for the low-calcium silicon–aluminum material is relatively slow, leading to extended setting times and reduced early-age strength. Consequently, the incorporation of BA, which has a lower calcium oxide content, resulted in a significant reduction in the strength of PSCMs, especially at 3 days. However, when the water glass content was 7%, both Mixtures BA0 and BA15 met the strength requirements for 42.5-grade Portland Phosphorus slag cement specified in JC/T 740-2006 [25], with the exception of the 3-day flexural strength, which was slightly below the threshold (not less than 3.5 MPa).

**Figure 5:**
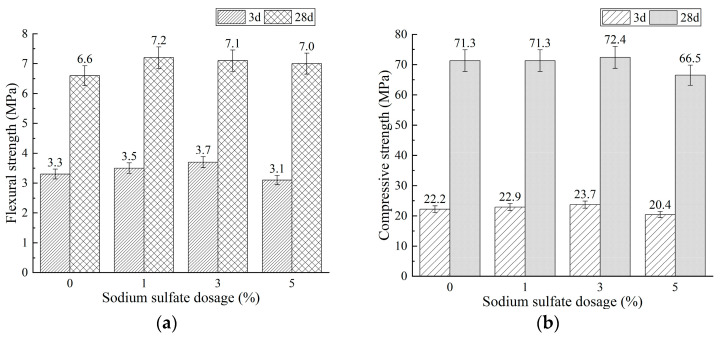
Effect of sodium sulfate (SS) dosage on strength of mixture BA0-WG7: (**a**) flexural strength and (**b**) compressive strength.

Additionally, as can be observed from Figure 4 and Figure 5, the flexural and compressive strengths of PSCMs exhibit distinct variation trends with increasing WG dosages. Specifically, the compressive strength increases steadily as the WG content rises, whereas the flexural strength initially increases and subsequently decreases. This divergence can be attributed to the different sensitivities of compressive strength and flexural strength to microstructural characteristics; the compressive strength is primarily influenced by overall compactness, while the flexural strength is more sensitive to the uniformity of pore distribution [61]. At low WG contents, the enhanced geopolymerization reaction generates additional hydration products, thereby increasing the overall compactness of the hardened paste and promoting both compressive and flexural strengths. However, when the WG content exceeds a critical threshold, the microstructural uniformity deteriorates, leading to uneven pore distribution and a reduction in flexural strength.

#### 3.1.2. Effect of the Sodium Sulfate Dosage

**Figure 6:**
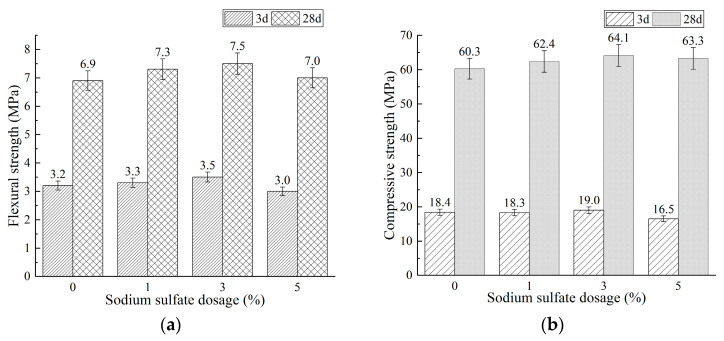
Effect of sodium sulfate (SS) dosage on the strength of mixture BA15-WG7: (**a**) flexural strength and (**b**) compressive strength.

Sodium sulfate (SS) was introduced as an additional activator to enhance the early-age flexural strengths of Mixtures BA0-WG7 and BA15-WG7, both of which contain 7% water glass (as detailed in Table 2). The effects of varying dosages (1%, 3%, and 5%) of SS on the compressive and flexural strengths of these mixtures were examined. The results are shown in Figure 5 and Figure 6.

As shown in Figure 5a and Figure 6a, the addition of sodium sulfate (SS) enhanced the flexural strengths of mixtures BA0-WG7 and BA15-WG7. Specifically, for mixture BA0-WG7, the 3-day flexural strength increased from 3.3 MPa (without SS) to 3.5 MPa and 3.7 MPa with the addition of 1% and 3% SS, respectively, representing increases of 6.1% and 12.1%. The 28-day flexural strength also rose from 6.6 MPa (without SS) to 7.2 MPa and 7.1 MPa. For mixture BA15-WG7, the 3-day flexural strength increased from 3.2 MPa (without SS) to 3.3 MPa and 3.5 MPa with 1% and 3% SS, while the 28-day flexural strength increased from 6.9 MPa (without SS) to 7.3 MPa and 7.5 MPa. However, a further increase in SS content to 5% resulted in a decrease in flexural strength for both mixtures. Additionally, Figure 5b and Figure 6b show that the effect of SS on the compressive strengths of mixtures BA0-WG7 and BA15-WG7 follows a similar trend to its effect on flexural strength, but with smaller changes in magnitude. This was because the composite activation of WG and SS accelerated the hydration rate of phosphorus slag-based materials (namely, mixture BA0-WG7 and BA15-WG7), thereby shortening their setting time. Additionally, besides C-A-S-H and N-A-S-H gels, a certain amount of monosulfate was also produced [61,62,63], which enhanced the early-age strength, particularly the flexural strength. However, when the SS dosage exceeded 3%, the paste exhibited rapid setting, affecting the morphology and uniformity of the hydration products, and adversely impacting the uniformity and porosity of the hardened paste, ultimately leading to a reduction in strength.

Figure 5 and Figure 6 demonstrate that mixtures BA0-WG7-SS3 and BA15-WG7-SS3 both comply with the compressive and flexural strength requirements stipulated in JC/T 740-2006 [25] for P.PS 42.5 Portland Phosphorous Slag Cement at both 3 and 28 days. To promote the utilization of diverse industrial solid wastes in low-carbon cementitious materials, subsequent experiments focus on the mixture BA15-WG7-SS3. We then investigate the regulation of setting time for this mixture, followed by the preparation of PSCM concrete. Subsequently, experimental studies evaluate the freeze–thaw resistance and sulfate resistance of the PSCM concrete.

### 3.2. Effect of Retarder Dosage on the Setting Time and Strength of PSCMs

**Figure 7:**
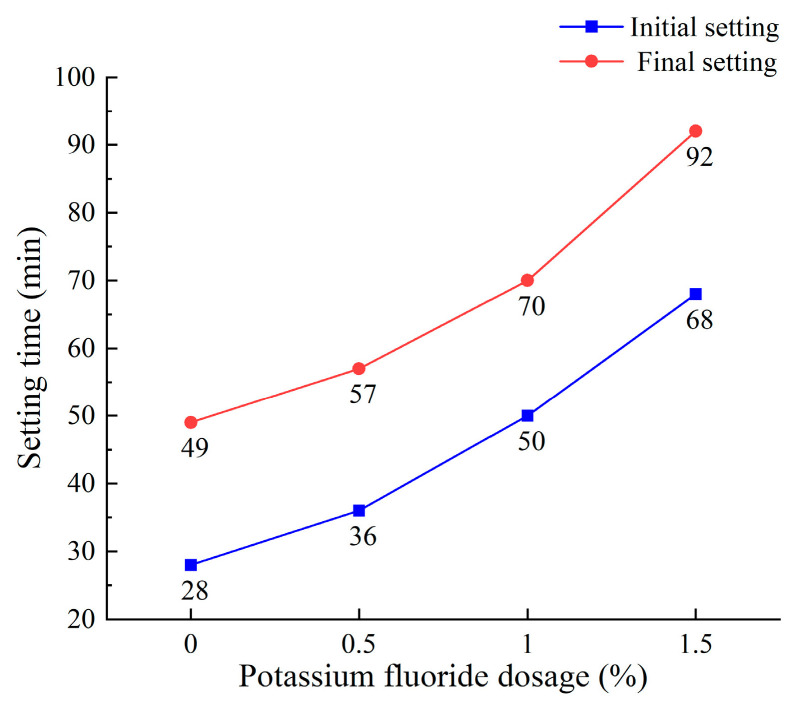
Effect of KF dosage on setting time of mixture BA15-WG7-SS3.

**Figure 8:**
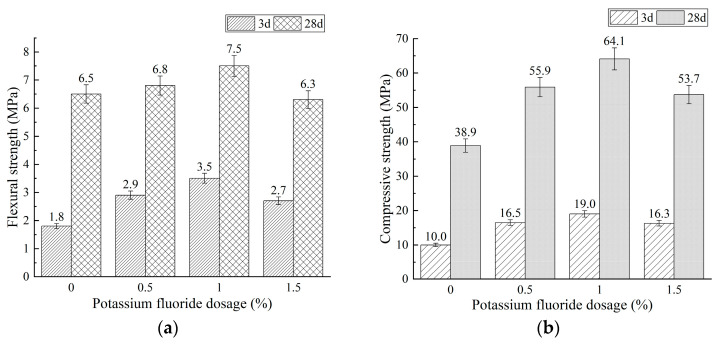
Effect of potassium fluoride dosage on strength of mixture BA15-WG7-SS3: (**a**) flexural strength and (**b**) compressive strength.

During the investigation of the effect of sodium sulfate dosage on mixture BA15-WG7-SS3, it was observed that this PSCM mixture exhibits a relatively rapid setting time. To address this, potassium fluoride (KF) was introduced as a retarder to adjust the setting time. This section studies the effects of varying KF dosages on the initial and final setting times, as well as the compressive and flexural strengths of the mixture. The results are summarized in Figure 7 and Figure 8.

As shown in Figure 7, the setting time of PSCM increased with a higher KF dosage. This is due to the reaction between fluoride ions (from KF) and calcium ions (from PS, BA, and L) during the early stages of hydration, leading to the formation of CaF2, which has extremely low solubility. The deposition of CaF2 on the surface of unhydrated PSCMs particles inhibits their further dissolution and subsequent hydration, thereby prolonging the setting time [64]. As the KF dosage increases, this inhibitory effect becomes more pronounced, allowing KF to effectively regulate the setting time of PSCMs. However, excessive KF dosages (e.g., 1.5%) result in a significant retarding effect, thereby reducing the strength of PSCMs (Figure 8).

Figure 8 indicates that as the KF dosage increases from 0% to 1.0%, both the flexural and compressive strengths of mixture BA15-WG7-SS3 gradually improve. Specifically, at 3 days, the flexural strength rises from 1.8 MPa to 3.5 MPa, and the compressive strength increases from 10.0 MPa to 19.0 MPa; at 28 days, the flexural strength increases from 6.5 MPa to 7.5 MPa, and the compressive strength rises from 38.9 MPa to 64.1 MPa. However, when the KF dosage further increases to 1.5%, the PSCM strength begins to decline. At this point, the 28-day flexural and compressive strengths are 6.3 MPa and 53.7 MPa, respectively. This can be attributed to the influence of the retarder on the depolymerization–repolymerization–hardening process of AAMs [56,58,64]. Specifically, when the mixture BA15-WG7-SS3 is mixed with water, free silicon–oxygen tetrahedral units and aluminum–oxygen tetrahedral units, along with calcium ions, are derived via dissolution from phosphorus slag (PS) and fluidized bed combustion bottom ash (BA) under the combined activating effects of water glass and sodium sulfate. Subsequently, these ions undergo polycondensation reactions to form gel products such as C-A-S-H and N-A-S-H gels, which gradually harden.

In the absence of KF, the reaction rates at each stage are significantly faster, leading to a relatively porous microstructure of the formed products and consequently lower strength in the hardened paste (Figure 8, KF% = 0). However, with the incorporation of KF, insoluble CaF_2_ forms on the surfaces of cementitious material particles, inhibiting the dissolution of silicon–oxygen and aluminum–oxygen tetrahedra units in the precursor. This delays the formation and growth of products such as C-A-S-H and N-A-S-H gels, resulting in a more improved microstructure of the hydration products and a reduction in the total porosity of the hardened paste, thereby enhancing its strength. When the KF dosage is 1.0%, the strength of the hardened paste reaches its peak, with 3-day flexural and compressive strengths of 3.5 MPa and 19.0 MPa, respectively. However, when the KF dosage increases to 1.5%, the excessively strong retarding effect reduces the quantity of C-A-S-H and N-A-S-H gels, leading to a decline in the hardened paste strength. Nevertheless, compared to the samples lacking KF, here, the microstructure at 3 days and 28 days was more uniform and denser, resulting in higher strength.

Moreover, Figure 7 and Figure 8 demonstrate that at a KF dosage of 1.0%, such as in mixture BA15-WG7-SS3, meets the setting time and strength requirements specified in JC/T 740-2006 [25] for P.PS 42.5-grade Portland Phosphorous Slag Cement. Therefore, the optimal PSCM formulation was determined to be BA15-WG7-SS3-KF1.0, which was used to prepare a PSCM concrete that would be evaluated for freeze–thaw resistance and sulfate attack resistance.

### 3.3. Freeze–Thaw Resistance and Sulfate Attack Resistance of PSCM Concrete

#### 3.3.1. Freeze–Thaw Resistance

**Figure 9:**
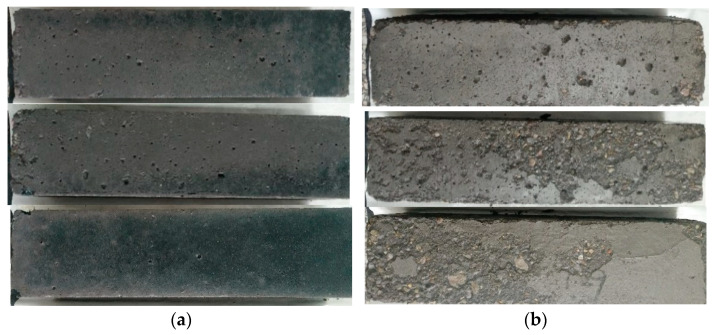
Appearance of PSCM concrete specimens after 175 freeze–thaw cycles: (**a**) PSCM-C50 and (**b**) OPC-C50 specimens.

The freeze–thaw resistance results of PSCM concrete are shown in Table 4. Figure 9 presents the appearances of PSCM-C50 and OPC-C50 specimens after 175 freeze–thaw cycles.

Table 4 shows that after 100 freeze–thaw cycles, although the mass loss rates of both PSCM-C30 and OPC-C30 specimens were below 5%, the relative dynamic elastic modulus of PSCM-C30 was 64.2%, while that of OPC-C30 decreased to 53.5% (below the threshold of 60%). This indicates that PSCM-C30 exhibited superior freeze–thaw resistance compared to OPC-C30, achieving resistance grades of F100 and F50, respectively. For C50 concrete, after 150 freeze–thaw cycles, the mass loss rates for PSCM-C50 and OPC-C50 were 0.08% and 1.24%, respectively, and their relative dynamic elastic moduli decreased to 62.8% and 68.3%, respectively. Both achieved a freeze–thaw resistance grade of F150. However, after extending the freeze–thaw cycles to 175 (Figure 9), although the mass loss rates of both specimens remained below 5%, the relative dynamic elastic modulus of OPC-C50 dropped to 56.6%, while that of PSCM-C50 remained above 61.1%. In conclusion, at the same strength grade, the PSCM concrete demonstrated a slightly better freeze–thaw resistance than the Portland cement concrete.

#### 3.3.2. Sulfate Attack Resistance

The sulfate attack resistance results for PSCM concrete are displayed in Table 5.

As shown here, after 30 cycles of dry–wet testing with a sodium sulfate solution, the compressive strength corrosion resistance coefficients for all concrete specimens, except OPC-C30, exceeded 100%. This indicates that during the initial stage of sulfate attack, these specimens exhibited higher compressive strength values compared to standard-cured specimens of the same age. However, as the numbers of dry–wet cycles increased to 60 and 90, the coefficients significantly decreased. This phenomenon is due to the simultaneous early-stage hydration of PSCMs and sulfate attack [23,47,49]. Initially, newly formed hydration and sulfate attack products such as monosulfate filled the pores of the hardened paste, improving pore structure and reducing porosity, thereby enhancing compressive strength. However, prolonged erosion and intensified attack led to an accumulation of erosion products, resulting in micro-crack formation and propagation within the concrete matrix, which adversely affected the sample’s mechanical properties [18,24].

Furthermore, Table 5 demonstrates that the corrosion resistance coefficients for PSCM-C30 concrete were 99% and 85% after 60 and 90 dry–wet cycles, respectively, while that for OPC-C30 was only 71% after 60 cycles (below the threshold of 75%). This indicates that the sulfate resistance grade of PSCM-C30 is KS90, whereas that of OPC-C30 is rated at KS30. Similarly, for PSCM-C50 and OPC-C50, the corrosion resistance coefficients were 103% and 74%, respectively, after 90 dry–wet cycles, indicating that the sulfate resistance grade of PSCM-C50 is higher than that of KS90, while that of OPC-C50 is rated at KS60. These results suggest that, at the same strength grade, PSCM concrete exhibited significantly higher sulfate resistance than Portland cement concrete. This enhanced performance may be attributed to the activating effect of sulfate on PSCMs.

### 3.4. Microstructural Investigation on PSCM

#### 3.4.1. XRD Analysis

Based on the aforementioned findings, the optimal proportion of PSCMs was selected to investigate hydration hardening behavior and microstructural characteristics. Moreover, three specific PSCM compositions, namely, mixtures BA0-WG7-KF1, BA0-WG7-SS3-KF1, and BA15-WG7-KF1, were additionally chosen. By systematically analyzing the effects of the presence or absence of BA and SS on the hydration processes and microstructural features of PSCM, the intrinsic relationships between material composition, microstructural characteristics, and macroscopic properties (e.g., mechanical strength and durability) of PSCMs were thoroughly explored.

**Figure 10:**
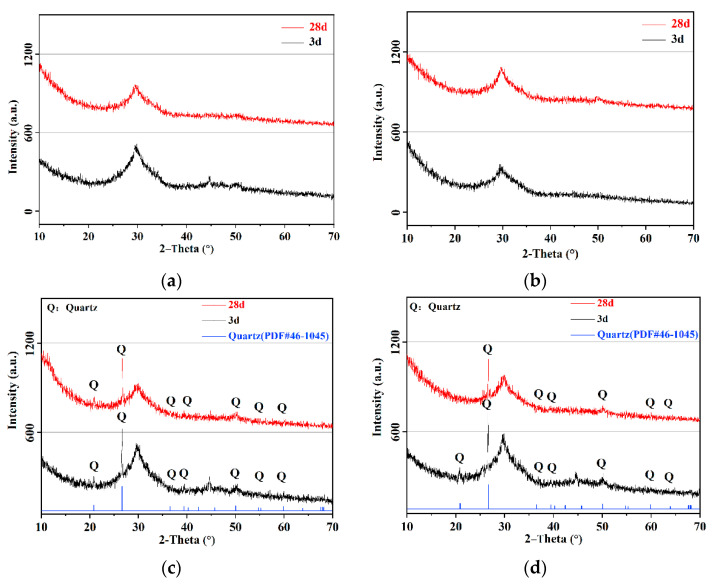
XRD patterns of paste samples from the selected PSCM compositions: (**a**) BA0-WG7-KF1, (**b**) BA0-WG7-SS3-KF1, (**c**) BA15-WG7-KF1, and (**d**) BA15-WG7-SS3-KF1.

Figure 10 illustrates the XRD patterns of samples derived from the pastes of mixtures BA0-WG7-KF1, BA0-WG7-SS3-KF1, BA15-WG7-KF1, and BA15-WG7-SS3-KF1, as listed in Table 2.

As shown in Figure 10, the four PSCM compositions display a broad diffraction peak within the 25° to 35° range of their XRD patterns. Additionally, diffraction peaks corresponding to quartz crystals were observed in the XRD patterns of the two PSCM compositions containing fluidized bed combustion bottom ash (BA) (Figure 10c,d), while no other crystalline phases were detected. Based on the chemical compositions of PS and BA (Table 1), as well as the XRD analysis results (Figure 1), it can be inferred that the broad diffused peak between 25° and 35° originated from incompletely hydrated PS, while the quartz crystals were introduced by BA. These findings indicate that the main hydration products of alkali-activated PSCMs are amorphous substances, specifically C-A-S-H and N-A-S-H gels, with no significant crystalline phases detected. This is consistent with the subsequent SEM analysis results presented in Section 3.4.4. It can also be seen in Figure 10 that the intensities of diffraction peaks within the range of 25–35° in the XRD patterns for these four PSCM compositions at 28 days are weaker compared to those of the 3 day samples. This observation suggests that PS is progressively consumed as the curing time extends. This consumption facilitates the formation of additional amorphous hydration products, thereby significantly enhancing the strength of the hardened paste. Furthermore, comparing Figure 10a with Figure 10b and Figure 10c with Figure 10d reveals that the addition of sodium sulfate does not significantly alter the types of main products in the PSCMs.

#### 3.4.2. FTIR Analysis

**Figure 11:**
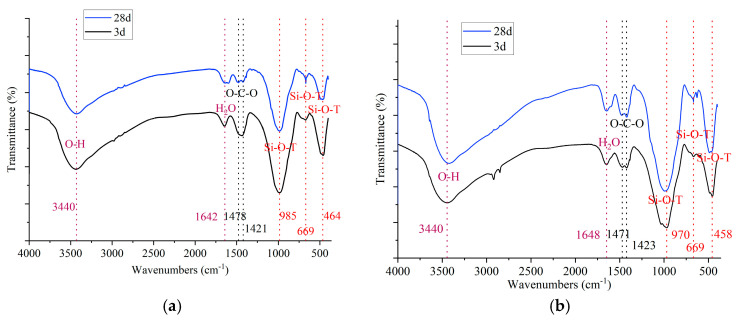

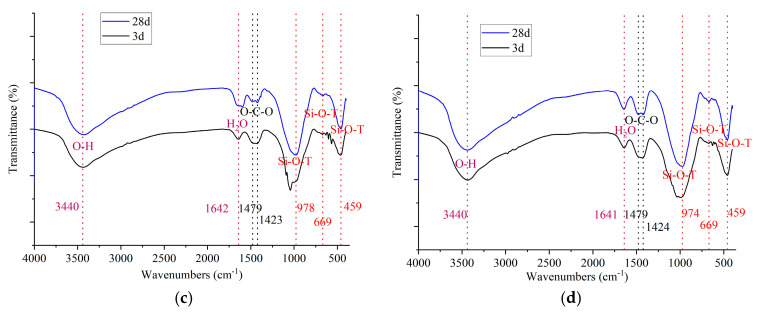
FTIR spectra of the selected PSCM compositions: (**a**) BA0-WG7-KF1, (**b**) BA0-WG7-SS3-KF1, (**c**) BA15-WG7-KF1, and (**d**) BA15-WG7-SS3-KF1.

Figure 11 presents the FTIR spectra of the selected PSCM samples, cured for 3 days and 28 days. The consistency of the spectra across all samples indicates that the incorporation of fluidized bed combustion bottom ash (BA) or sodium sulfate has minimal impact on the primary hydration products in water glass-activated PSCMs during both early and late hydration stages.

In the FTIR analysis of cementitious materials, vibrational absorption bands with wavenumbers below 1000 cm^−1^ primarily correspond to silicates and carbonates in the hydration products, while those above 1600 cm^−1^ are typically attributed to chemically bound water [65,66,67,68,69,70,71,72,73,74]. Specifically, the bands at approximately 460 cm^−1^, 670 cm^−1^, and 975 cm^−1^ are associated with the vibration of Si–O–T bonds (where T represents tetrahedral Si or Al), the bands at 1422 cm^−1^ and 1479 cm^−1^ correspond to the vibration of O–C–O bonds in CO_3_^2−^, and the bands at 1640 cm^−1^ and 3440 cm^−1^ are attributed to chemically bound water. The vibrational absorption bands corresponding to Si–O–T, O–C–O, and H–O–H bonds are identified in the spectra (Figure 11).

As shown in Figure 11, two broad and intense bands at approximately 459 cm^−1^ and 978 cm^−1^, along with a weak band at 669 cm^−1^, are observed in the spectra of all samples. These bands are attributed to the bending, asymmetric stretching, and symmetric stretching vibrations of Si–O–T bonds, respectively [70,71,72]. The presence of these characteristic vibration bands indicates the formation of C-A-S-H and N-A-S-H gels in the hydration products of the PSCM compositions. Moreover, the intensity of the two broad intense bands at approximately 459 cm^−1^ and 978 cm^−1^ increases with curing age, suggesting increases in the contents of C-A-S-H and N-A-S-H gels over time. Meanwhile, the band at 669 cm^−1^ becomes sharper as curing progresses, indicating the enhanced structural ordering of the C-A-S-H and N-A-S-H gels with prolonged curing. These findings regarding the increased quantity and improved structural orderliness of C-A-S-H and N-A-S-H gels provide a compelling explanation for the age-dependent strength gain in the four PSCM mixtures (Figure 5, Figure 6 and Figure 8).

The bands at 1642 cm^−1^ and 3440 cm^−1^ are assigned to the bending and stretching vibrations of the chemically bonded H–O–H in the hydration products of the samples [65,69,73], which may be imparted by the presence of C-A-S-H and N-A-S-H gels or monosulfate. Additionally, the spectra of all samples exhibit characteristic bands at approximately 1423 cm^−1^ and 1479 cm^−1^. These bands are attributed to the asymmetric stretching vibrations of O–C–O bonds in the CO_3_^2−^ ions present in different polymorphs of CaCO_3_ (calcite and vaterite) [65,66,67,68,70,71,72,73]. These polymorphs are likely formed as a result of the carbonation of hydration products during the preparation of powder samples for FTIR analysis. Nevertheless, calcium carbonate crystals were not detectable by XRD in these samples (Figure 10), which may indicate either low crystallinity or a small amount.

#### 3.4.3. TG-DTG Analysis

**Figure 12:**
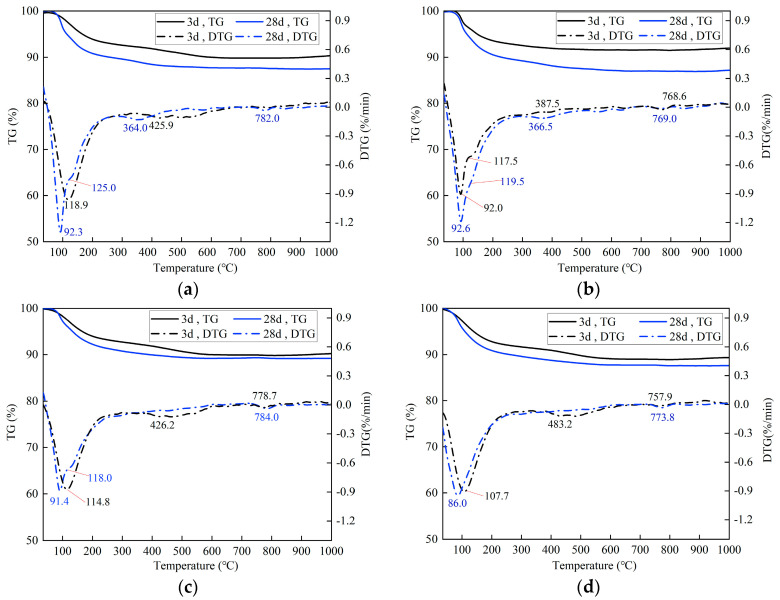
TG-DTG curves of the selected PSCM compositions cured for 3 days and 28 days: (**a**) BA0-WG7-KF1, (**b**) BA0-WG7-SS3-KF1, (**c**) BA15-WG7-KF1, and (**d**) BA15-WG7-SS3-KF1.

Thermogravimetric analysis was performed to examine the hydration products of the selected PSCM compositions. The thermogravimetric (TG) and derivative thermogravimetric (DTG) curves for these compositions after curing for 3 days and 28 days are presented in Figure 12.

The result of thermogravimetric analyses of alkali-activated slag and Portland cement samples are discussed in References [71,75,76,77,78]. In this paper, the broad endothermic mass loss peak observed below 200 °C in the DTG curve of PSCM paste samples can be attributed to water adsorbed into or bound to the binder to varying degrees [71,77]. Specifically, mass loss peaks at approximately 90 °C are attributed to the removal of free water present in the pore network of the binder or adsorbed from the atmosphere, while mass loss peaks (or shoulders) at about 120 °C are attributed to the dehydration of hydration products, including monosulfate phases (AFm) and C-A-S-H and N-A-S-H gels. The intensity of this broad mass loss peak increases with prolonged curing time, indicating that the ongoing hydration reaction of PSCM results in the formation of more hydration products, such as C-A-S-H and N-A-S-H gels and AFm. This finding is consistent with the FTIR analysis results (Figure 11).

Moreover, the mass loss peaks at approximately 365 °C in the DTG curves of compositions BA0-WG7-KF1 and BA0-WG7-SS3-KF1 cured for 28 days can be attributed to the dehydration of C-A-S-H and N-A-S-H gels [72,78]. In contrast, the mass loss peaks at about 425 °C in the DTG curves of samples BA0-WG7-KF1, BA15-WG7-KF1, and BA15-WG7-SS3-KF1 cured for 3 days are attributed to the dehydroxylation of portlandite, which originates from the hydration of lime. Notably, the absence of a mass loss peak due to portlandite in the DTG curves of the three afore-mentioned compositions cured for 28 days, as well as mixture BA0-WG7-SS3-KF1 cured for both 3 days and 28 days, is evident. This absence is likely due to the consumption of portlandite during the continuous hydration of PSCMs and the carbonation of portlandite during the preparation of powder samples for TG analysis. Additionally, the DTG peaks at approximately 780 °C in all curves of the PSCM samples cured for 3 days and 28 days are attributed to the decarbonization of calcium carbonates.

#### 3.4.4. SEM-EDS Analysis

**Figure 13:**
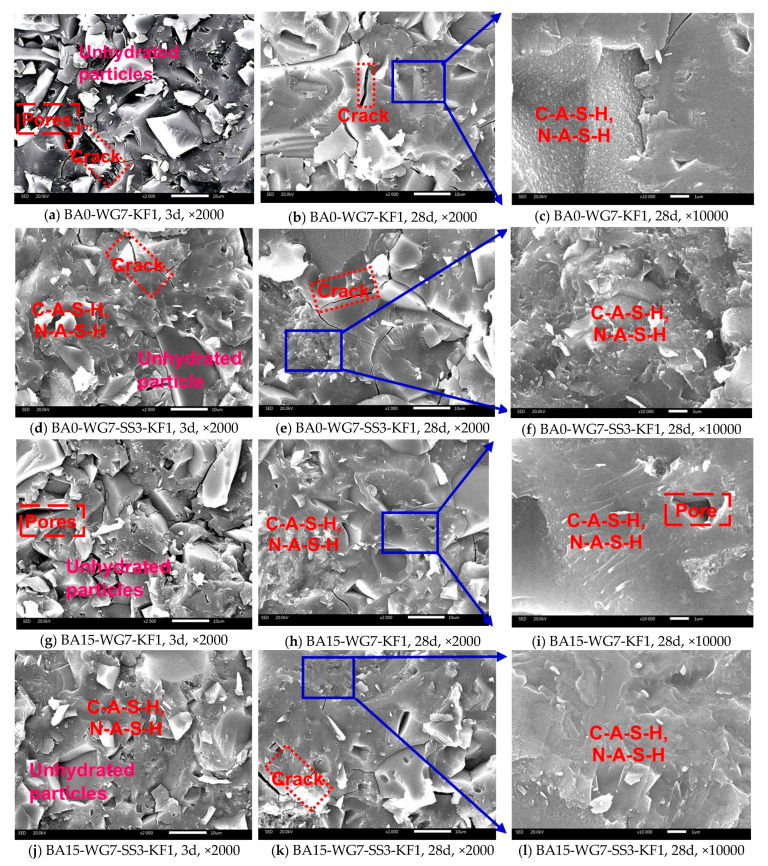
SEM images of selected PSCM paste specimens at different ages.

The morphologies of the hydration products and microstructures of selected PSCM paste samples cured for 3 days and 28 days were investigated using SEM. The corresponding SEM images are shown in Figure 13.

As illustrated in Figure 13a,d,g,j, the 3-day PSCM samples exhibit a microstructure characterized by cracks, pores, and unhydrated cementitious material particles wrapped in C-A-S-H and N-A-S-H gels. No portlandite (CH) crystals were detected in the SEM images of these samples, likely due to the low CH content at this early stage, which renders them undetectable. By 28 days, the hardened PSCM paste showed a significant reduction in cracks and pores, resulting in a more compact microstructure. This improvement can be attributed to the ongoing hydration as the curing period extended, leading to the substantial consumption of PS, BA, and CH, and the formation of additional C-A-S-H and N-A-S-H, as confirmed by FTIR and TG-DTG analyses. The resulting hydration products reduce porosity, refine the microstructure, and enhance the mechanical properties of the hardened paste. Additionally, no calcium aluminate hydrate crystals were observed in any PSCM samples, including those incorporating sodium sulfate (e.g., BA0-WG7-SS3-KF1 and BA15-WG7-SS3-KF1), where no ettringite or gypsum crystals were found. These findings are consistent with those of XRD, FTIR, and TG analyses.

**Figure 14:**
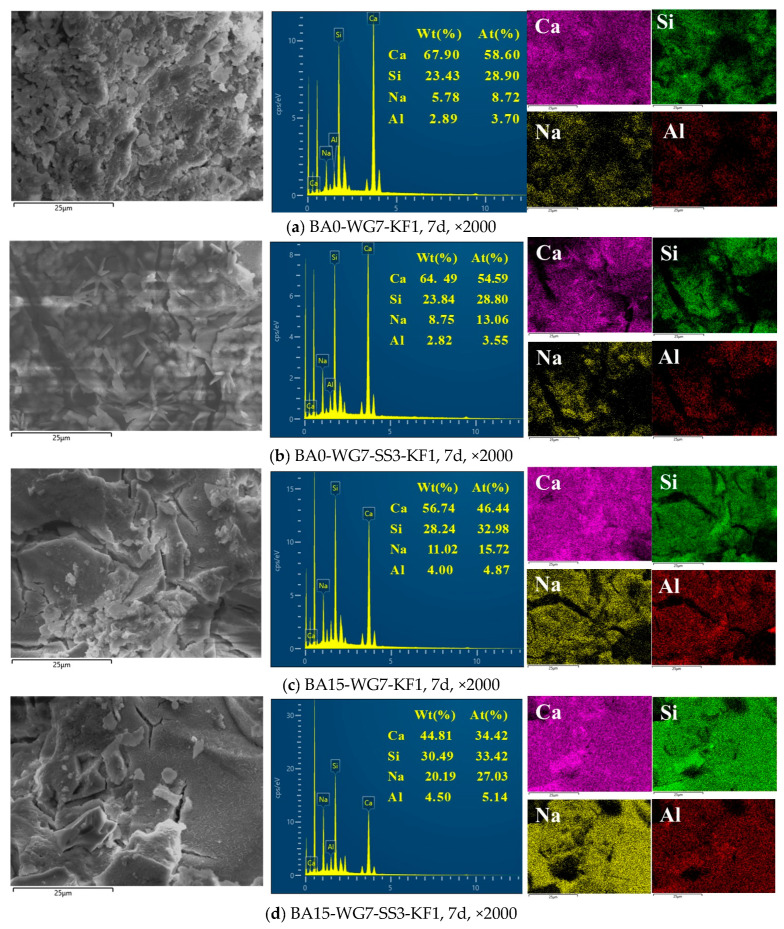
SEM-EDS analysis results for the hydration samples of the selected PSCM at 7 days of age.

Figure 14 presents the elemental distribution within the hydration samples of the selected PSCM at 7 days of age, as determined by SEM-EDS analyses.

As illustrated in Figure 14, the hydration samples of the selected PSCM exhibited high concentrations of calcium (Ca) and silicon (Si), along with trace amounts of aluminum (Al) and sodium (Na). Based on the results presented in the figure, the molar ratios of calcium, aluminum, and sodium to silicon in each hydration sample of the selected PSCMs were calculated. The detailed data are systematically summarized in Table 6.

As shown in Table 6, the incorporation of SS resulted in a decrease in the calcium-to-silicon molar ratio (Ca/Si) from 2.03 to 1.90 (samples BA0-WG7-KF1 and BA0-WG7-SS3-KF1). Conversely, the sodium-to-silicon molar ratio (Na/Si) increased from 0.30 to 0.45 (samples BA0-WG7-KF1 and BA0-WG7-SS3-KF1). Similarly, for the BA15-WG7-KF1 sample, the incorporation of SS led to a reduction in the Ca/Si molar ratio from 1.41 to 1.03 (samples BA15-WG7-KF1 and BA15-WG7-SS3-KF1), as well as an increase in the Na/Si molar ratio from 0.48 to 0.81 (samples BA15-WG7-KF1 and BA15-WG7-SS3-KF1). These findings confirm that the incorporation of SS leads to a decrease in the Ca/Si ratio and an elevation in the Na/Si ratio within the hydration products of PSCMs.

It can also be seen in Table 6 that the incorporation of BA resulted in a marked decrease in the Ca/Si ratio of the hydration products of PSCMs, while both the Na/Si ratio and the Al/Si ratio exhibited significant increases. Specifically, after introducing BA, the Ca/Si ratio in sample BA0-WG7-KF1 decreased from 2.03 to 1.41 (sample BA15-WG7-KF1), while the Na/Si ratio increased from 0.30 to 0.48 and the Al/Si ratio increased from 0.13 to 0.15 (samples BA0-WG7-KF1 and BA15-WG7-KF1), respectively. Similarly, following the addition of BA, the Ca/Si molar ratio in sample BA0-WG7-SS3-KF1 decreased from 1.90 to 1.03 (sample BA15-WG7-SS3-KF1), whereas the Na/Si ratio increased from 0.45 to 0.81 and the Al/Si ratio increased from 0.12 to 0.15 (samples BA0-WG7-SS3-KF1 and BA15-WG7-SS3-KF1), respectively. These findings indicate that the incorporation of BA effectively promotes a reduction in the Ca/Si molar ratio, as well as increases in both the Na/Si and Al/Si ratios within the hydration products of PSCMs.

The SEM-EDS analysis results demonstrate that the hydrated samples of the optimally formulated PSCMs exhibit a reduced Ca/Si ratio, along with increased Al/Si and Na/Si ratios. These observations may provide a plausible explanation for the improved mechanical properties and durability of PSCMs.

Based on the aforementioned microstructural analysis results, it is reasonable to infer that the predominant hydration products of the developed PSCMs are C-A-S-H and N-A-S-H gels, with no detectable crystalline phases such as ettringite, gypsum, or calcium aluminate hydrates present. The absence of these crystalline phases significantly contributes to the material’s pronounced shrinkage and the prevalence of microcracks in its microstructure. Future research will focus on an in-depth investigation of the mechanisms governing PSCM shrinkage control.

### 3.5. Cost Analysis for the Optimal Formulation of PSCMs

Based on the market prices of each component [79], a cost analysis was performed for the optimal PSCM formula (BA15-WG7-SS3-KF1), with the results presented in detail in Table 7.

As shown in Table 6, the total cost of the optimal PSCM formulation is approximately CNY 385 per ton, which is substantially lower than that of Grade 42.5 ordinary Portland cement (CNY 440 per ton). This highlights the significant cost advantages of PSCMs when used in practical applications.

## 4. Conclusions

A type of clinker-free cementitious material, namely, phosphorus slag-based cementitious material (PSCM), was prepared through alkali activation using industrial solid waste, including granulated electric furnace phosphorus slag (PS) and fluidized bed combustion bottom ash (BA), along with lime (L) as a precursor component. Initially, the effects of varying dosages of alkaline activators, including water glass and sodium sulfate, as well as retarder (potassium fluoride, KF), on the properties (setting time and strength) of PSCM were investigated to determine the optimal formulation. Subsequently, the sulfate resistance and freeze–thaw resistance of PSCM concrete prepared using this optimal formulation were evaluated. Finally, the hydration and microstructure of selected PSCM compositions were characterized using X-ray diffraction (XRD), Fourier transform infrared spectroscopy (FTIR), thermogravimetry (TG), and scanning electron microscope (SEM). The following conclusions have been drawn:

(a) When the mass ratio of precursor components for PSCM, i.e., granulated electric furnace phosphorus slag (PS), fluidized bed combustion bottom ash (BA), and lime (L), is 80:15:5, the optimal dosages of water glass (WG), sodium sulfate (SS), and retarder (KF) are 7%, 3%, and 1%, respectively. The designed PSCM exhibits an initial setting time of 50 min and a final setting time of 70 min, with flexural strengths of 3.5 MPa at 3 days and 7.5 MPa at 28 days, and compressive strengths of 19.0 MPa at 3 days and 64.1 MPa at 28 days. These setting times and strength values comply with the requirements for P.PS 42.5-grade Phosphorous Slag Portland Cement specified in the Chinese standard JC/T 740-2006 [25];
(b) The durability evaluation results indicate that PSCM concrete prepared using the optimized PSCM formulation exhibited superior sulfate resistance and freeze–thaw resistance compared to ordinary Portland cement (OPC) concrete of the same strength grade. Under the test conditions employed in this study, the sulfate resistance grades of PSCM-C30 and PSCM-C50 both exceeded KS90, whereas those of OPC-C30 and OPC-C50 were KS30 and KS60, respectively. Additionally, the freeze–thaw resistance grades of PSCM-C30 and OPC-C30 were F100 and F50, respectively, while those of PSCM-C50 and OPC-C50 were F175 and F150, respectively
(c) The microstructural investigation demonstrates that the primary hydration products of the designed PSCM are calcium aluminosilicate hydrate (C-A-S-H) and sodium aluminosilicate hydrate (N-A-S-H) gels, with no detectable crystalline phases such as ettringite, gypsum, or calcium aluminate hydrates. FTIR and TG-DTG analyses confirm that the hydration reaction of PSCM continues to progress as the curing time increases, leading to the formation of additional C-A-S-H and N-A-S-H gels. SEM observations reveal that the microstructure of the hardened PSCM paste becomes progressively denser with extended curing time. This can be attributed to the ongoing hydration reaction of PSCM, which results in the formation of additional hydration products, thereby significantly improving the microstructure of the hardened paste, and enhancing both its mechanical strength and its durability.

## Figures and Tables

**Table 1:** Chemical composition of phosphorus slag (PS), fluidized bed combustion bottom ash (BA), and L (wt. %).

	SiO_2_	Al_2_O_3_	Fe_2_O_3_	CaO	MgO	K_2_O	Na_2_O	SO_3_	P_2_O_5_	Loss on Ignition
PS	38.09	3.85	0.33	43.64	1.98	3.05	0.37	1.27	4.21	0.52
BA	55.74	27.52	3.85	2.26	1.59	1.36	0.51	1.35	0.22	3.69
L	0.44	0.18	0.07	93.4	0.95	/	/	0.79	/	4.10

**Table 2:** Formulations of PSCM mixtures.

Mixture No.	Cementitious Component (%)			Activator ^a^		Retarder ^a^ (i.e., KF) (%)
	PS	BA	Lime (L)	Water Glass (WG) (Na_2_O wt. %)	Sodium Sulfate (SS) (wt. %)	
BA0-WG5	95	0	5.0	5.0	0	1.0
BA0-WG6	95	0	5.0	6.0	0	1.0
BA0-WG7	95	0	5.0	7.0	0	1.0
BA0-WG8	95	0	5.0	8.0	0	1.0
BA15-WG5	80	15	5.0	5.0	0	1.0
BA15-WG6	80	15	5.0	6.0	0	1.0
BA15-WG7	80	15	5.0	7.0	0	1.0
BA15-WG8	80	15	5.0	8.0	0	1.0
BA0-WG7-SS1	95	0	5.0	7.0	1.0	1.0
BA0-WG7-SS3	95	0	5.0	7.0	3.0	1.0
BA0-WG7-SS5	95	0	5.0	7.0	5.0	1.0
BA15-WG7-SS1	80	15	5.0	7.0	1.0	1.0
BA15-WG7-SS3	80	15	5.0	7.0	3.0	1.0
BA15-WG7-SS5	80	15	5.0	7.0	5.0	1.0
BA15-WG7-SS3-KF0	80	15	5.0	7.0	3.0	0.0
BA15-WG7-SS3-KF0.5	80	15	5.0	7.0	3.0	0.5
BA15-WG7-SS3-KF1	80	15	5.0	7.0	3.0	1.0
BA15-WG7-SS3-KF1.5	80	15	5.0	7.0	3.0	1.5

^a^ The dosages of admixtures, including activator and retarder, are expressed as percentages of the total mass of PS, BA, and L.

**Table 3:** The mix proportions of PSCM concrete.

Concrete No.	W/B	Mix Design (kg/m^3^)			Activator ^c^		Retarder ^c^
		PSCM	Sand	Crushed Stone	Water Glass (WG) (Na_2_O wt. %)	Sodium Sulfate (SS) (wt. %)	KF (wt. %)
PSCM-C30	0.44	333 ^a^	746	1121	7	3	1
PSCM-C50	0.33	500 ^a^	578	1122	7	3	1
OPC-C30	0.44	333 ^b^	746	1121	0	0	0
OPC-C50	0.33	500 ^b^	578	1122	0	0	0

^a^ The mass ratio of the components in PSCM, namely, PS, BA, and L, is 80:15:5. ^b^ Ordinary Portland cement concrete of strength grades C30 and C50 (OPC-C30 and OPC-C50), prepared using P·O 42.5 Portland cement, served as the reference groups in this study. This cement, supplied by China Huaxin Cement Co., Ltd. (Wuhan, China), exhibited compressive strengths of 31.4 MPa at 3 days and 47.5 MPa at 28 days. ^c^ The dosages of admixtures, including activator and retarder, are expressed as a percentage of the mass of the PSCM.

**Table 4:** Freeze–thaw resistance results of PSCM concrete.

Concrete No.	Mass Loss of Specimens After *n* Freeze–Thaw Cycles, Δ*W_n_*/%					Relative Dynamic Elastic Modulus of Specimens After *n* Freeze–Thaw Cycles, *P*/%				
	0	50	100	150	175	0	50	100	150	175
PSCM-C30	0	0	0.5	/	/	100	88.1	64.2	/	/
PSCM-C50	0	0	0	0.08	0.10	100	91.2	83.1	68.3	61.1
OPC-C30	0	0.4	1.7	/	/	100	79.1	53.5	/	/
OPC-C50	0	0	0.1	1.24	2.07	100	96.2	81.7	62.8	56.6

**Table 5:** Sulfate attack resistance results of PSCM concrete.

Concrete No.	Compressive Strength Corrosion Resistance Coefficient of PSCM Concrete After *n* Cycles of Dry–Wet Testing with Sodium Sulfate Solution, K_f_ (%)		
	30	60	90
PSCM-C30	110	99	85
PSCM-C50	121	112	103
OPC-C30	93	71	/
OPC-C50	104	95	74

**Table 6:** Molar ratios of calcium, aluminum, and sodium to silicon in the hydrated samples of the selected PSCMs.

	Molar Ratios	Ca/Si	Na/Si	Al/Si
The Selected PSCM				
BA0-WG7-KF1		2.03	0.30	0.13
BA0-WG7-SS3-KF1		1.90	0.45	0.12
BA15-WG7-KF1		1.41	0.48	0.15
BA15-WG7-SS3-KF1		1.03	0.81	0.15

**Table 7:** Cost analysis of the optimal PSCM formulation developed in this study.

	Components	PSCM						P·O 42.5
Item		PS	BA	L	WG (by Na_2_O)	SS	KF	
Mass percentage, %		80	15	5	13.6 ^a^	3.0	1.0	100
Component price [79], CNY/ton		160	100	400	550	860	12,000	440
Component Cost, CNY		128	15	20	74.6	25.8	120	440
Total cost, CNY/ton		383.4						440

^a^ The mass percentage of WG is calculated based on its Na_2_O mass fraction and the optimal Na_2_O dosage specified in the PSCM formulation.

## Data Availability

The original contributions presented in this study are included in the article. Further inquiries can be directed to the corresponding author.
